# Numerical simulation study on distinguishing nonlinear propagation regimes of femtosecond pulses in fused silica

**DOI:** 10.1038/s41598-024-56460-0

**Published:** 2024-03-09

**Authors:** Faqian Liu, Tingting Xi, Lanzhi Zhang, Dongwei Li, Zuoqiang Hao

**Affiliations:** 1 Shandong Provincial Engineering and Technical Center of Light Manipulations & Shandong Provincial Key Laboratory of Optics and Photonic Devices, School of Physics and Electronics, Shandong Normal University, Jinan, 250358 China; 2 School of Physical Sciences, University of Chinese Academy of Sciences, Beijing, 101407 China; 3 Collaborative Innovation Center of Light Manipulation and Applications, Shandong Normal University, Jinan, 250358 China; 4 Joint Research Center of Light Manipulation Science and Photonic Integrated Chip of East, China Normal University and Shandong Normal University, East China Normal University, Shanghai, 200241 China

**Keywords:** Ultrafast photonics, Supercontinuum generation

## Abstract

We perform numerical simulations to investigate the nonlinear propagation dynamics of femtosecond Gaussian and vortex beams in fused silica. By analyzing the extent of spectral broadening, we are able to distinguish between the linear, self-focusing, and filamentation regimes. Additionally, the maximum intensity and fluence distribution within the cross-section of the vortex beams are analyzed for different incident laser energies. The results demonstrate a direct correlation between the spectral broadening and the peak intensity of the femtosecond laser pulse. As a result, this provides a theoretical foundation for distinguishing different propagation regimes, and determining critical powers for self-focusing and filamentation of both femtosecond Gaussian and structured beams.

## Introduction

The nonlinear propagation of intense ultrashort laser pulses in transparent media has attracted significant attention in ultrafast science. Many interesting phenomena are associated with the propagation, such as self-focusing effect^1^, spectral broadening^2^, light bullets generation^3^, and filamentation^4^. Depending on incident laser power, the propagation processes typically involve linear, self-focusing, and filamentation, which can be elucidated as follows: when the laser pulse has a peak power higher than the critical power for self-focusing (commonly denoted as P_cr_), the self-focusing of the laser beam surpasses the diffraction effect, resulting in an intensification of the laser beam until ionization occurs^5^. Once the plasma generated reaches a sufficiently high density to counterbalance the Kerr effect, the laser pulse undergoes a defocusing process, thus initiating filamentation. In the case of well-developed filamentation, the laser intensity reaches a clamped state, commonly known as the intensity clamping effect^6,7^. Understanding the propagation dynamics of ultrashort pulses in different media is crucial for optimizing their performance in various applications. Extensive experimental investigations and simulations have explored the nonlinear propagation processes associated with self-focusing, clamped intensity, multiple filamentation in the case of Gaussian pulses^8–13^. However, there is still a lack of research focusing on the nonlinear propagation dynamics of ultrashort structured beams.

The nonlinear propagation dynamics of structured beams are also crucial both for fundamental physics studies and practical applications, such as laser micromachining^14^, laser spectroscopy^15^, air lasing^16^, and optical waveguide^17^. However, the nonlinear propagation characteristics of femtosecond vortex beams which have a donut-shaped intensity profile, differ significantly from those of Gaussian beams^18–20^. Specifically, when an intense femtosecond vortex beam propagates in optical media, it undergoes a unique nonlinear evolution. Under the effect of self-focusing, the beam will split into multiple hot spots surrounding the central singularity of the vortex beam. Subsequently, if the incident laser power exceeds the critical power for self-focusing, these hot spots collapse into multiple filaments. In our recent study, the nonlinear propagation regimes of self-focusing and filamentation of femtosecond vortex laser pulses in fused silica have been experimentally distinguished by measuring the spectral broadening of the laser beam^21^. This technique, which we refer to as the S-scan method, has proven to be effective in characterizing the propagation dynamics of femtosecond beams. Building upon this, in this paper, we conduct numerical simulations to elucidate the underlying physical mechanism of the S-scan method. Our results clearly demonstrate that the blue-shift of the cut-off wavelength is mostly driven by the maximum peak intensity during self-focusing and filamentation processes of the laser pulse.

## Method

To simulate the propagation of the femtosecond laser pulse in the fused silica, we solve the nonlinear Schrödinger Equation (NLSE), coupled with electron generation^22^, which can be expressed as follows:

$${\partial }_{z}E=\frac{i}{2{k}_{0}}{T}^{-1}{\nabla }_{\perp }E+iDE+i\frac{{\omega }_{0}}{c}{n}_{2}T{\int }_{-\infty }^{t} \mathcal{R}\left(t-{t}^{{{\prime}}}\right){\left|E\left({t}^{{{\prime}}}\right)\right|}^{2}d{t}^{{{\prime}}}E$$

1 $$- i\frac{{k_{0} }}{{2n_{0} \rho_{c} }}T^{ - 1} \rho E - \frac{{\beta^{\left( \kappa \right)} }}{2}\left| E \right|^{2\kappa - 2} E - \frac{\sigma }{2}\rho E,$$

2 $$\frac{\partial \rho }{{\partial t}} = \frac{{\beta^{\left( \kappa \right)} \left| E \right|^{2\kappa } }}{{U_{i} }} + \frac{{\sigma \rho \left| E \right|^{2} }}{{U_{i} }} - \frac{\rho }{{\tau_{rec} }},$$

3 $${\mathcal{R}}\left( {t - t^{\prime}} \right) = \left( {1 - \alpha } \right)\delta \left( {t - t^{\prime}} \right) + \alpha \frac{{1 + \omega_{R}^{2} \tau_{R}^{2} }}{{\omega_{R} \tau_{R}^{2} }}e^{{ - \frac{{t - t^{\prime}}}{{\tau_{R} }}}} sin\left( {\omega_{R} \left( {t - t^{\prime}} \right)} \right),$$

where $E$ is the electric field envelope, z is the propagation distance, ${k}_{0}=2\pi /{\lambda }_{0}$ is the central wavenumber of the laser pulse with a central wavelength ${\lambda }_{0}$=800 nm. ${\nabla }_{\perp }=\frac{{\partial }^{2}}{\partial {{\text{x}}}^{2}}+\frac{{\partial }^{2}}{\partial {{\text{y}}}^{2}}$ describes the transverse diffraction, and the corresponding operator ${\text{T}}=1+\left(\frac{{\text{i}}}{{\upomega }_{0}}\right)\partial {\text{t}}$ describes spatio-temporal focusing effect.${\text{D}}={\sum }_{{\text{n}}\ge 2}\left(\frac{{{\text{k}}}^{\left({\text{n}}\right)}}{{\text{n}}!}\right){\left({\text{i}}\partial {\text{t}}\right)}^{{\text{n}}}$ describes dispersion with coefficient of ${k}^{(n)}={\left.\frac{{\partial }^{n}k}{\partial {\omega }^{n}}\right|}_{{\upomega }_{0}}$. The third term accounts for the Kerr response with a coefficient of ${n}_{2}=3.2\times 1{0}^{-16}{cm}^{2}/W$. The operator ${\text{T}}$ before this term represents the self-steepening effect. The Kerr responsibility is described by the function $\mathcal{R}\left(t-{t}^{{{\prime}}}\right)$, which is expressed in Eq. (3) with parameters: $\alpha$=0.18, vibrational delay time ${\tau }_{R}$=32 fs, and resonance frequency ${\omega }_{R}$=0.082 fs^-1^. The fourth term accounts for the electron defocusing effect where the critical density of the plasma ${\uprho }_{{\text{c}}}=1.73\times 1{0}^{21}\mathrm{ c}{{\text{m}}}^{-3}$. The fifth term represents the multiphoton absorption, with ionization coefficient of ${\upbeta }^{(\upkappa )}=8.4\times 1{0}^{-67}{{\text{cm}}}^{9}/ {W}^{5}$ for $\upkappa =6$, and the band-gap energy of ${{\text{U}}}_{{\text{i}}}=9\mathrm{ eV}$. The last term accounts for the collision ionization absorption, with the inverse Bremsstrahlung cross section $\sigma =6.57\times {10}^{-19} {{\text{cm}}}^{2}$. The corresponding ionization effects are considered in Eq. (2). The electronic recombination process is also considered with the response time ${\tau }_{rec}=150\mathrm{ fs}$. The initial electric field envelopes of the Gaussian and vortex laser pulses can be written as.

4 $$E = E_{0} \exp \left( { - r^{2} /2w^{2} - t^{2} /0.72\tau_{0}^{2} - ik_{0} r^{2} /2f} \right),$$

5 $$E = E_{0} \frac{r}{w}\exp \left( { - r^{2} /2w^{2} - t^{2} /0.72\tau_{0}^{2} - ik_{0} r^{2} /2f + im\varphi } \right),$$

respectively, where the beam radius w = 0.13 mm for Gaussian, and 0.06 mm for vortex beam, respectively. The lens has a focal length of f = 15 mm. $\varphi$ is the azimuthal angle. The pulse duration (FWHM) is τ = 65 fs.

## Results and discussion

**Figure 1:**
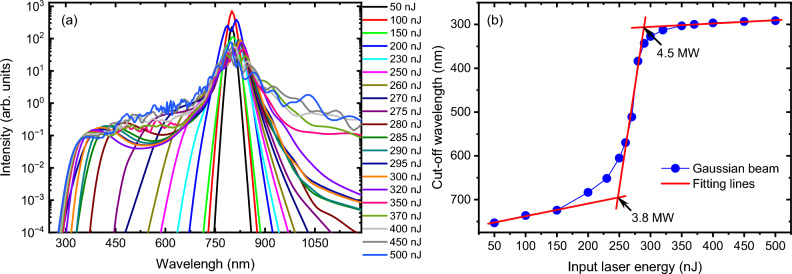
(**a**) The far-field spectrum of a Gaussian laser beam for different input laser energies, (**b**) the evolution of the cut-off wavelength as a function of input laser energy.

First, we investigate the spectral evolution of a femtosecond Gaussian beam for various input laser energies. The laser pulse’s spectrum is calculated by applying Fourier transformation to the laser pulse, integrating across the entire laser beam. The input laser energy is gradually increased from a very low energy of 50 nJ to a relatively high energy of 500 nJ. The far-field spectra for various input laser energies are plotted in Fig. 1a. As the input laser energy increases, the spectrum becomes broader, notably exhibiting significant broadening on the blue side. At a very low input laser energy of 50 nJ, a slight broadening of the spectrum is observed compared to the fundamental laser spectrum. When the laser energy is further increased from 50 to 230 nJ, the spectral broadening is predominantly symmetric. Subsequently, as the laser energy is increased from 230 to 285 nJ, the blue-side spectrum becomes increasingly broader. As the laser energy continues to increase, the blue-side spectral broadening gradually approaches a saturated state, forming a typical laser filamentation-induced supercontinuum spectrum. To quantitatively evaluate spectral changes as a function of laser energy, similar to our previous experiment^21^, we determine the blue-side cut-off wavelength. The cut-off wavelength is determined as the wavelength where the spectral intensity is equal to 10^–3^. It is plotted in Fig. 1b. It is evident that the cut-off wavelength undergoes a gradual extending with increasing incident laser energy, followed by a rapid transition towards saturation. This trend is in excellent agreement with the results obtained in experiment^21^. Based on this trend, three distinct regimes can be easily distinguished, corresponding to linear propagation, self-focusing, and filamentation processes. In order to determine the critical points for these three processes, linear fittings are made on the data. The intersections of the fitted lines provide the deviation positions of 247.7 nJ and 289.6 nJ, which correspond to the self-focusing critical power of 3.8 MW and the mature filamentation critical power of 4.5 MW, respectively. These values of self-focusing and filamentation critical powers are slight smaller than the experimental ones^21^, which may be attributed to differences in the initial parameters used for the simulation.

It is important to note that the obtained 3.8 MW crossover power does not represent the nominal critical power for self-focusing, as the specific value of the crossover power can vary depending on experimental conditions^20,23–27^^.^ Moreover, directly determining the exact nominal critical power in experiment can be challenging^26^. However, for the purpose of providing clear and referenceable measurements for experimental investigations and practical applications of femtosecond filamentation, in this study, we adopt the crossover power as the critical power for self-focusing, as done in our recent work and relevant literatures^10,11,20,21,24,28,29^.

**Figure 2:**
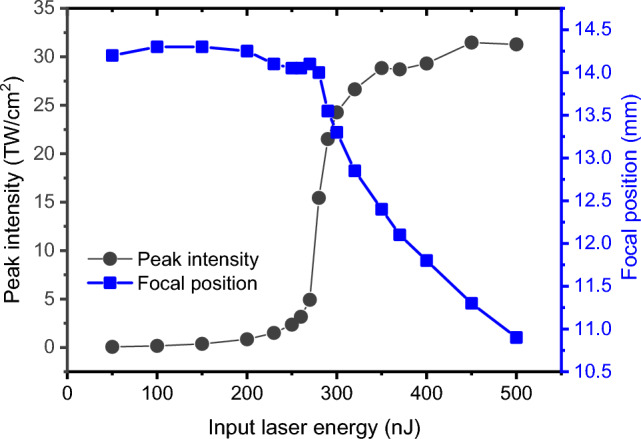
The evolution of maximum intensity and corresponding focal position of the Gaussian beam for different laser energies.

Previous research has demonstrated that the maximum blue-side extension of the supercontinuum spectrum is highly sensitive to changes in the peak intensity within the filamentation^7^. Moreover, the saturation of the maximum blue shift is attributed to the intensity clamping in the filamentation process. In our previous study, we experimentally demonstrated that the blue-side spectral broadening observed during the laser filamentation is primarily influenced by the laser intensity^30^. Thus, we plot the maximum intensity of the laser pulse for different input laser energies in Fig. 2. It is apparent that the trend of peak intensity in relation to laser energy is consistent with that of cut-off wavelength shown in Fig. 1b. By comparing and analyzing these two figures, we can derive insights into the characteristics of laser pulse propagation as the input laser energy increases, as well as the underlying physical mechanisms. When the input laser energy is relatively low, the peak intensity of the laser pulse is very weak and the medium does not experience ionization^31^. Under this condition, the broadening of the laser spectrum only comes from the self-phase modulation. The spectrum exhibits symmetric broadening on both red and blue sides, and the amount of spectral broadening increases roughly linearly with the input laser energy. In this stage, the laser experiences nonlinear effects of the self-focusing effect and self-phase modulation. This stage can be then referred to as the self-focusing regime. As the input laser energy continues to increase, the pulse undergoes spatiotemporal self-focusing, leading to a rapid increase in the peak intensity and subsequent ionization of the medium. Many nonlinear effects are involved and the filamentation occurs. Consequently, the laser spectrum experiences significant broadening, particularly on the blue side. This stage represents the transition from self-focusing to mature filamentation. As the input energy further increases, the intensity within the filamentation gradually reaches a constant value, indicating the state of laser intensity clamping. This saturation is also observed in the spectral broadening on the short wavelength side. In this stage, the filament eventually transforms into a mature filament, a phenomenon consistent with the results in Ref.^7^. This stage can be then referred to as the filamentation regime. Additionally, we also observe that even when the laser intensity has reached the saturation, the blue-side spectrum exhibits a slight broadening as the input laser energy is further increased. This can be attributed to two factors. First, with an increase in input energy, the filamentation length will also increase. Our previous research has shown that a longer length of the laser filament leads to higher conversion efficiency in supercontinuum generation^30^. This means that more of the laser energy is converted into a broader range of wavelengths. Consequently, the spectral intensity in the vicinity of the wavelength where the cut-off wavelength is determined also increases, leading to a slight broadening of the spectrum. The second factor should be related to the intensity clamping, which refers to the phenomenon where the intensity in the laser filamentation reaches a maximum value and no longer increases with further increase in incident laser energy. However, this clamping intensity value should not be a fixed constant and will slightly increase with higher incident laser energy. This also will contribute to the further broadening of the spectrum. Furthermore, the curve also exhibits two deviation positions that correspond to the critical powers for self-focusing and filamentation, respectively. From these observations, we can then conclude that the extension of the cut-off wavelength is closely related to the peak intensity of the laser pulse, forming the fundamental basis for the S-scan method.

For comparison, the critical power for self-focusing is further studied by using the commonly used focus-shifting method^8^. The focal position is determined by analyzing the maximum intensity along the propagation of the laser pulse. The relationship between the focal position and the input laser energy is plotted in Fig. 2 (blue line with solid squares). It is evident that for relatively lower laser energies, there is no significant change in the focal position. However, with a further increase in laser energy, the focal position shifts quickly towards the focusing lens. This evolution tendency is consistent with previous studies^8,10,11^, and serves as the basis for using the focus-shifting method to determine the critical power for self-focusing. The transition position (~ 280 nJ) can be regarded as the position where the pulse’s peak power is equal to the critical power for self-focusing in fused silica^8^. However, we can see that the detailed nonlinear propagation process of the laser pulse cannot be further distinguished by using the focus-shifting method.

**Figure 3:**
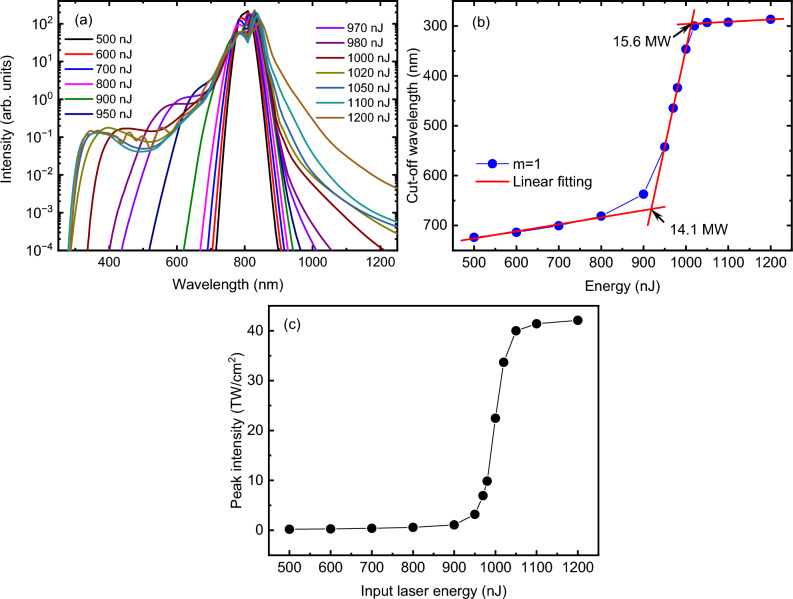
(**a**) Spectrum and (**b**) corresponding cut-off wavelength of the vortex laser beam for different input laser energies. (**c**) The laser peak intensity for different input laser energies. The vortex beam has a topological charge of 1.

The S-scan method is further applied to investigate the behavior of femtosecond vortex laser beams. Figure 3a shows the spectra of the vortex beam with a topological charge of m = 1 for different laser energies. Similar to the case of Gaussian laser pulses, the spectrum exhibits progressive broadening as the input laser energy increases, with a notable extension towards the blue side. The cut-off wavelengths of the spectra are determined using the same criterion (the wavelength at which the spectral intensity is equal to 10^–3^) and are plotted in Fig. 3b. The change in the cut-off wavelength with the input laser energy exhibits a similar trend to that observed for Gaussian beam, characterized by three distinct regions. Further analysis is conducted by performing segmented linear fitting to obtain the critical powers for self-focusing and filamentation. The first intersection point of the fitted lines occurs at approximately 918.26 nJ, corresponding to the self-focusing critical power of approximately 14.1 MW. Similarly, the second intersection point is observed at approximately 1012.9 nJ, corresponding to the critical power for mature filamentation, calculated to be approximately 15.6 MW. Similar to the case of Gaussian beam, the peak intensities for various vortex laser energies are plotted in Fig. 3c, exhibiting a remarkably similar trend to the cut-off wavelength. This observation further validates the conclusion that the variation of the cut-off wavelength with increasing input laser energy is primarily influenced by the peak intensity of the laser.

**Figure 4:**
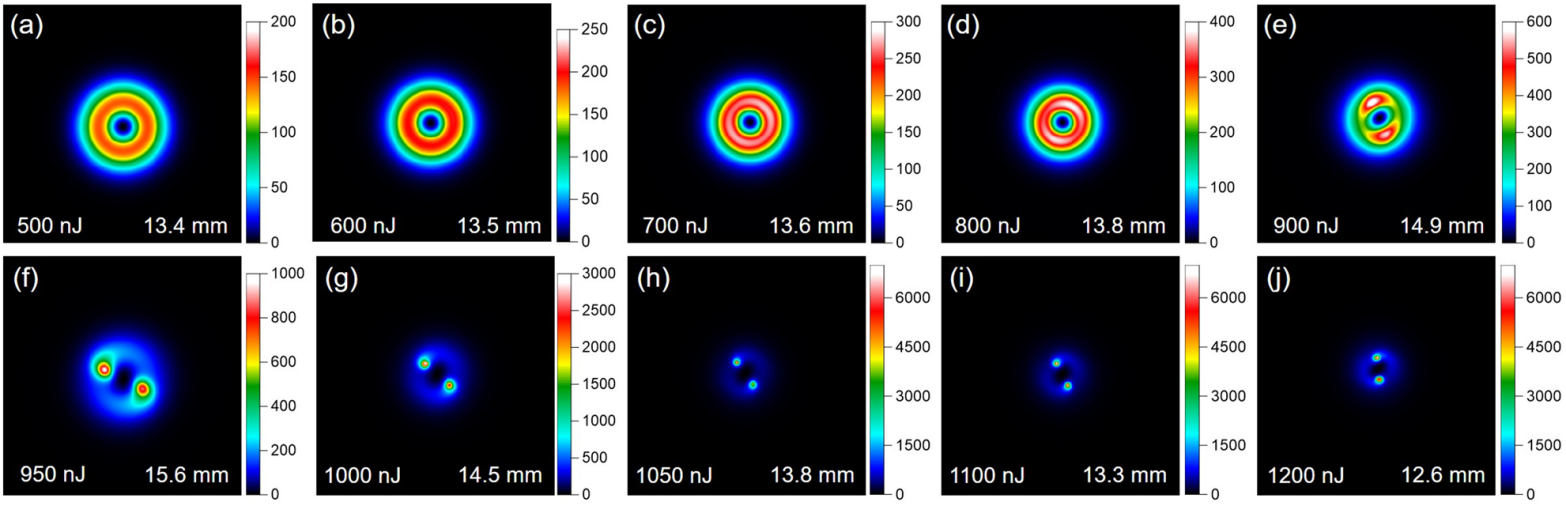
The vortex laser fluence distributions at typical propagation distances for typical input laser energies.

Finally, Fig. 4 presents the fluence distributions of the vortex laser beam at typical propagation distances for several input laser energies. At relatively low levels of input laser energy, the laser beam maintains a ring distribution with a singularity in the center, as shown in Fig. 4a–c. As the input laser energy increases, the laser beam undergoes a gradual collapse, resulting in the formation of relatively weak filaments under the effect of the self-focusing (see Fig. 4d–f). When the laser energy exceeds 1050 nJ, two distinct filaments emerge within the ring structure, and their fluences remain relatively constant, indicating the formation of mature filaments. The distribution patterns provide valuable insights into the changes in laser propagation characteristics as the input energy is increased. Specifically, the transition from self-focusing to filamentation regime can be easily identified as the laser energy gradually increases from 900 to 1050 nJ. This result is highly consistent with those obtained using the S-scan method (Fig. 3), thereby further confirming the reliability of the S-scan technique. Furthermore, a notable difference in the azimuth angle of these two filaments can be observed from these laser fluence distributions. The difference is due to the twist of vortex laser beam, which will induce helical filamentation^32^. Therefore, the filaments position will be different for different propagation distances, as shown in Fig. 4.

## Conclusions

In conclusion, we numerically demonstrate the effectiveness of the S-scan method in distinguishing different regimes of nonlinear propagation of femtosecond Gaussian and vortex pulses in fused silica. Specifically, the linear, self-focusing, and filamentation regimes can be successfully distinguished, which is consistent with experimental findings. Importantly, our results reveal that the changes in cut-off wavelengths are primarily attributed to the maximum laser intensity. Our study provides valuable insights into femtosecond pulses evolution. This is significant for accurately estimating the nonlinear propagation processes of femtosecond laser pulses at different input laser energies. It also helps in determining the critical powers for self-focusing and mature filamentation of both femtosecond Gaussian and structured beams. Additionally, an independent study conducted by Kinyaevskiy et al. has presented compelling evidence that supports the effectiveness and validity of the S-scan method^21,31^.

## Data Availability

The datasets generated and analyzed during the current study are available from the corresponding author on reasonable request.
